# Issues arising following a referral and subsequent wait for extraction under general anaesthetic: impact on children

**DOI:** 10.1186/1472-6831-15-3

**Published:** 2015-01-17

**Authors:** Michaela Goodwin, Caroline Sanders, Gill Davies, Tanya Walsh, Iain A Pretty

**Affiliations:** The Dental Health Unit, School of Dentistry, The University of Manchester, Williams House, Manchester Science Park, M15 6SE Manchester, England; Centre for Primary Care, The University of Manchester, Williamson Building, Oxford Road, Manchester, M13 9PL England; Public Health England, Greater Manchester Centre, 3 Piccadilly Place, Manchester, M1 3BN England

**Keywords:** Caries, Children, Pain, Impact, General anaesthetic

## Abstract

**Background:**

Untreated caries in young children can result in a referral for extraction in hospital under general anaesthetic (GA). This study aims to explore the impact of caries during the ensuing wait for GA on children resident in the North West of England.

**Methods:**

The study involved 456 respondents referred to six hospitals in the Northwest of England. Over a two-month period each of these children/ families completed a questionnaire and gave permission to access their referral and consultation notes.

**Results:**

Children (6.78 years old: 1.50 to 16.42) had on average five teeth extracted (ranging from one to a full clearance, with all teeth removed). Sixty seven per cent of parents reported their child had been in pain, 26% reported schools days being missed and 38% having sleepless nights. The average time from referral to operation was 137 days. Results indicated that children could be in discomfort during their wait, as pain was experienced, on average, 14 days before the operation. Wait time significantly predicated the number of sleepless nights *b* = .004, *t*(340) = 2.276, *p* = .023.

**Conclusions:**

It is clear that pain, sleepless nights and missed school are a feature during a wait for dental GA and can be exacerbated by an extended wait. These data support the need for not only effective prevention of caries within primary care to reduce wait times and experience of GA but also effective management of pain and infection during a prolonged wait for treatment.

## Background

Gross, untreated caries in children can result in significant morbidity, including those elements described within the PUFA index: pulplal involvement, ulceration, fistula and abscess [1, 2]. These effects are not only observed short term, but can persist with long-lasting impacts on both oral and general health in later life [3]. Whilst caries has been shown to contribute to problems including weight, communication difficulties and impaired cognitive development, it can also impact on day-to-day activities, for example attending school and sleeping at night [1, 4–6].

When dental pain or discomfort experienced by a child becomes too severe (typically due to the presence of infection) a common treatment choice is extraction. General anaesthetic (GA) has been a technique used within dentistry for over 100 years as a way of carrying out dental treatment in young children, anxious adults and individuals with special care needs. In England the use of GA has been restricted to a hospital setting following the publication of the Poswillo report [7] and then 'A Conscious Decision' [8].

While GA extraction is frequently agreed to be the only option in cases involving very young children, it should be carefully considered against other options such as Local Anaesthetic or Inhalation Sedation due to the risks associated with any GA procedure. A number of studies have confirmed the negative effects of dental GA (DGA) on both the child and their immediate family, indicating that associated morbidity can be an upsetting experience for not only the patient but also the parents who care for them [9]. This is important to consider in relation to the repercussion on future dental attendance and treatment, for example the impact of DGA on dental anxiety. Anxiety may be the reason a child requires a GA, either through fear of treatment under local anaesthetic or apprehension (of the child or parent) causing a delay in seeking early (and regular) treatment at a general dental practice [10–12]. Given that general anaesthetic has also shown an association with anxiety post procedure [9], this could create a cycle of negative response with continuing dental anxiety and a pattern of reoccurring attendance for dental GA. Therefore the problems associated with dental decay could be exacerbated when considered alongside the prolonged wait and subsequent treatment using GA.

While DGA can be distressing it is acknowledged as a vital component of dental care and has been shown to produce immediate benefits in regard to oral health [13]. DGA is of particular benefit when other treatment options are unsuitable particularly in regards to those with medical problems, which restrict treatment options or young children where options for treatment can be limited due to cooperation.

### Aim

This study aims to explore the impact of decay and its sequela on children awaiting extractions under GA, which referring or triaging dentists have deemed are beyond simple restoration.

### Hypothesis

1. There will be a significant difference observed between hospitals and the time children wait from the decision to refer until treatment (RTT)
2. Increased wait times will correlate with increased negative impacts on children

## Methods

This was an observational study that recruited patients who attended six randomly selected hospitals throughout the North West of England for extractions under GA. Hospitals were selected using a random number generator. Each hospital was then visited for a period of two months with the researcher attending every session scheduled for DGAs for extraction, commonly known as outpatient GA, i.e. those not specifically for special needs or other complex procedures. This permitted the authors to gain a representative sample from each hospital, characterising the number of patients seen on average within each site. Sample size was calculated based on information gained from both the Dental Observatory [14] (now the Dental Public Health Intelligence Programme) and from a previous service evaluation completed by the authors. Based on these data and given the ratio of male to female participants, an absolute precision of 5% and a 95% confidence level, a sample of 374 children was calculated to be required [15]. Other proportions with known data were calculated i.e. proportion of children seen who were five years and younger, but as the ratio for male to female was almost 50:50 this yielded the largest minimum sample size needed.

In addition to information collected from the referral and clinical notes, a questionnaire was given to parents with questions on impact of the situation based on the Children’s Dental Health Survey [16] with additional questions on school attendance and effects on sleep added following further research [17, 18]. Full ethical approval for this study was obtained from the NRES Committee North West Preston (11/NW/0503) and all participants gave informed consent before taking part.

## Results

Given the final sample size of 456 there were deemed sufficient numbers for further analysis around descriptive statistics. Data were entered into SPSS (IBM Version 20) and these observational data were analysed using appropriate methods, taking into account assumptions for parametric tests.

All analyses for this paper have been carried out on those who consented to participate. Therefore the unit of analysis are the individual children attending for extraction under GA. In total 493 participants attended and underwent a DGA procedure and 456 agreed to complete the questionnaire resulting in a 93% consent rate. However this figure was out of a potential 606 available places on the GA list, which were not all filled due to ‘Failure to Attend’ (FTA) or patients sent home due to illness or having eaten shortly before the operation. The total FTA rate observed within the study was 16%.

Analysis on referral data was only carried out where notes were present for that participant. At hospital 4 the majority of complete referral letters were missing. However often the medical history and teeth requested for extraction were recorded from the referral notes in the main hospital procedure notes, and these data were collected (Table 1).

**Table 1:** **Basic demographics and (referral) reported impact of decay on children**

	Hospital 1 n = 117	Hospital 2 n = 48	Hospital 3 n = 35	Hospital 4 n = 76	Hospital 5 n = 49	Hospital 6 n = 130	Total = 456
Gender	64/53 55%45%	22/26 45%54%	19/17 53%47%	38/38 50%50%	26/22 54%46%	75/55 58%42%	244/211 54%46%
Male/Female							
Age: Mean	6.36 (1.5-13.17)	6.54 (2.17-13.83)	7.56 (3.83-13.92)	6.78 (1.83-13.42)	7.01 (3.67-12.42)	6.95 (1.75-16.42)	6.78 (1.5-16.42)
(Min–Max)							
Pain* Yes	55 (47%)	12 (25%)	17 (49%)	3 (17%)	32 (82%)	49 (43%)	168 (45%)
Anxiety* Yes	32 (28%)	9 (19%)	3 (9%)	1 (9%)	13 (33%)	19 (18%)	77 (22%)
Infection* Yes	35 (31%)	13 (27%)	13 (37%)	5 (26%)	29 (74%)	49 (43%)	144 (39%)
Teeth extracted: Mdn (Min–Max)	8 (1–20)	7 (1–15)	4 (1–7)	4 (1–12)	5 (1–13)	4 (1–18)	5 (1–20)
Wait time (days) Mdn (Min–Max)	239	141	81	-	126	82	137
	(6–577)	(24–217)	(43–217)		(6–362)	(1–418)	(1–577)

*Recorded by referring dentist. Mdn = Median.

Medical history was recorded in referral notes in 82% of cases, with 22% of these records reporting relevant medical history. The DGA lists included in this study were not specifically for special needs or complex care however those with reported medical issues included heart conditions, cerebral palsy, clefts, and various allergies, including penicillin. Special needs, behavioural difficulties or learning disabilities were also recorded. These factors could have contributed to the need for DGA when age or numbers of teeth to be extracted were not necessarily the main factor triggering the referral.

A history of previous DGA for that child was gained from the questionnaire. Those who reported their child had a previous extraction experience under general anesthetic ranged from 12% to 37% across the 6 hospitals (Table 2).

**Table 2:** **Previous DGA experiences**

	1	2	3	4	5	6	Chi square
No previous GA	103 (88%)	40 (85%)	26 (72%)	66 (87%)	31 (63%)	89 (70%)	*x* ^2^ = 24.216 (5),p = 0.0001
Previous DGA	14 (12%)	7 (15%)	10 (28%)	10 (13%)	18 (37%)*	39 (30%)*	

*To determine exactly which hospital is significantly different, hospitals were looked at individually against the collective (combining hospital data). Hospital 5 and 6 had significantly more children attending for repeat DGA than that of hospital 1,2,3 and 4.

**Figure 1:**
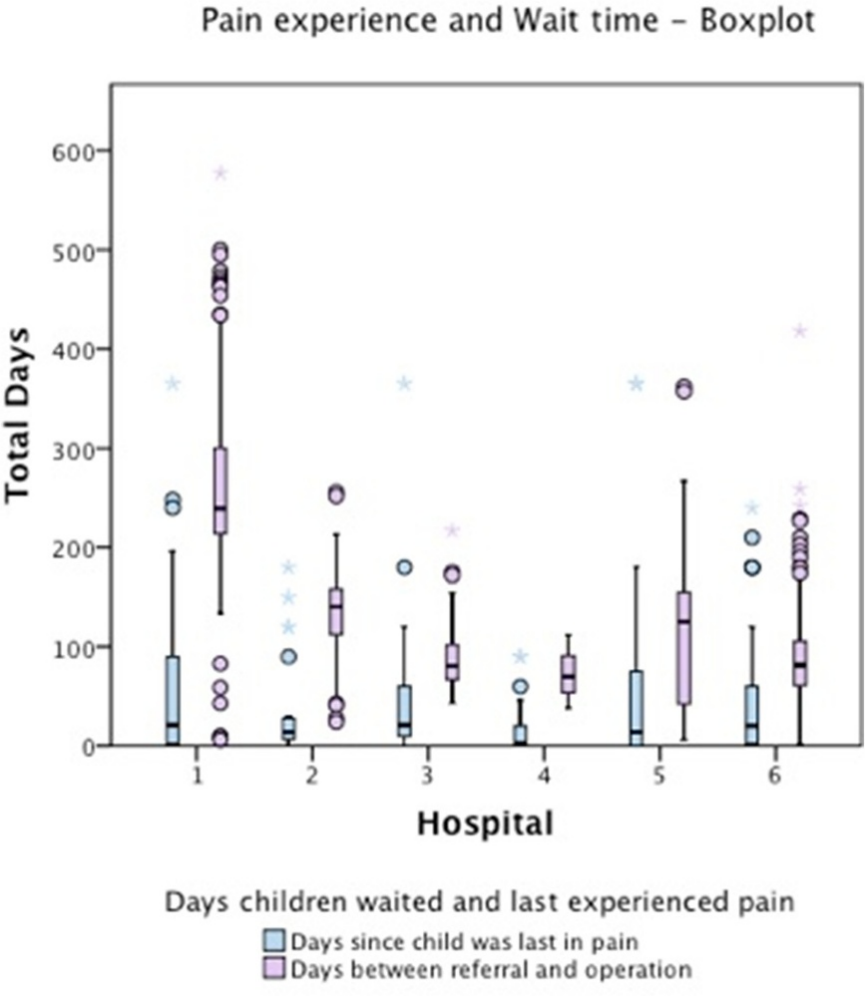
**Number of days since: pain last experienced and time taken to referral.** *Circles represent cases deemed to be outliers and stars represent extreme values*.

The duration of RTT was explored between hospitals using Kruskal Wallis because of the non normal distribution and violation of homogeneity of variance (Hospital 4 was removed from analysis as there was insufficient information available as referral letters were not present within the consultation notes). A significant difference was detected H (5) =170.117, p = 0.0001, the null hypothesis was rejected and pairwise multiple comparisons computed. Dunn-Bonferroni tests were undertaken to compare ranked data [19] and indicated children waited significantly longer from the time they were referred to the date of the operation at hospital 1 compared to all other hospitals (H1 vs. 2 U = 108.88, r = 0.497, H1 vs. 3 U = 162.02 r = .681, H1 vs. 5 U = 129.991 r = 0.575 H1 vs. 6 U = 161.415 r = 0.801 and for hospital 2 compared to hospital 6 U 52.527 r = 0.238) (medians presented in Figure 1).

Pain was experienced by two thirds of patients (67%) and over a third had sleepless nights (38%) (Table 3). Overall 78% of respondents stated they had at least one of the issues listed in relation to the dental problem they were referred for. There was no correlation between number of negative impacts and time a child waited for their operation (r_s_ = 0.049,p = 0.365).

**Table 3:** **Impact of dental problem on child, recorded by parents (n = 456)**

	Yes/No	Days median/mode (min - max)
Pain –days since child was in dental pain	(67%/33%)	**14**/0 (0–365 days)
Limitation on chewing or talking	(22%/78%)	-
Affect on self confidence	(4%/96%)	-
Impact on emotions (irritability)	(20%/80%)	-
Social behaviour i.e. playing and speaking	(6%/94%)	-
Absenteeism due to dental issue (days)*	(26%/74%)	**2**/2 (1–30 days)**
Sleepless nights due to dental issues	(38%/62%)	**3**/10 (1 - 10+ nights)**

*Filtered for those school age at operation.

**Filtered on those who experienced this impact.

The time taken from initial referral until the operation and the number of days since a child was in pain was not significantly correlated (spearman’s rho). This indicated that pain was not necessarily just experienced at referral and could continue sporadically throughout the wait, no matter how long the wait was (r_s_ = 0.130,p = 0.061). In fact 45% of patients had experienced pain within the last month (31 days) and 27% pain within the last 7 days. Given an average wait of 137 days (approximately 4.6 months); this confirmed children are in pain while they wait for their operation. This is substantiated by the affect on sleep and missed school throughout the wait time. Multiple regression indicated number of days from referral to operation significantly predicted sleepless nights *b* = .004, *t*(340) = 2.276, *p* = .023 (having adjusted for age, see Table 4). Therefore for every day a child had to wait they would miss an additional 0.004 night’s sleep.

**Table 4:** **Multiple regression analysis on wait time and impact on sleep**

	B	SE B	β	t	P value
Constant	2.002	0.504		3.969	
Wait time (from initial referral to operation)	0.004	0.002	-0.123	-2.276	0.023
Age	-0.12	0.005	0.125	2.306	0.022

*According to this model;*

A child referred to Hospital 1, where there is an average wait of 264 days, would experience 3.058 sleepless nights due to their dental problem (having adjusted for age); a child at Hospital 4, where there is an average wait of 73-days would experience 2.294 sleepless nights due to their dental problem (having adjusted for age).

Despite 25% missing days from school there were insufficient data to support a regression analysis.

Figure 1 shows a boxplot of wait times and last pain experienced for each hospital. While wait times differed across hospitals, with greater variability, the median time in days since last pain experienced remained relatively low, with pain consistently experienced within a short period of time before the operation occurred. In fact a substantial proportion of children (i.e. falling within the 75th percentile) were still experiencing pain before the majority of children had been seen for their operation. This is troubling as it suggests pain is experienced throughout the wait for GA.

Children who experienced no pain were explored further to understand why they may have been referred and if they could be treated in another way. Age and number of teeth extracted were not significantly different from the sample that experienced pain to those who did not. However, a statistically significant difference was found for those who were referred with a medical condition/indicator, for example behavioural problems or special needs. Thirty one per cent of those with no pain had medical indicators compared to just 18% of those referred with pain *x*^2^ = 6.204 (1), p = 0.013. Therefore those referred without experiencing pain may still require treatment under GA but for various medical/behavioural reasons and not just the severity of caries and pain/infection experienced.

RTTs were not normally distributed, thereby violating the assumption for parametric tests so non-parametric tests were used. There was no difference observed when looking at length of RTT and for those who reported any; pain (U = 14899.5 (349) p = 0.064), *any* days off from school (U = 10960.5, p = 0.957) or *any* sleepless nights (U = 14348.5, p = 0.648) using Mann Whitney U. This indicates sleepless nights, missed days of school and pain can be factors throughout the duration of the wait. Therefore a prolonged RTT is likely to cause a negative impact on a child’s day-to-day activities and emotional state.

## Discussion

This study aimed to explore the effects of increased RTT on children referred for a DGA extraction across 6 randomly selected hospitals in the North West. This is partly in response to current research indicating there are varying wait times and services available throughout England as a whole [20] and anecdotal evidence from dentists and commissioners as to the issues likely to be encountered with a prolonged wait for GA. The study examined the impact on those waiting for a DGA alongside information gained from referral notes.

Analysis of the RTT compared to when they were last in pain found these were not significantly correlated. This suggests children were not only in pain at the time of referral but could have been in pain throughout their wait. In fact almost half (45%) of those asked reported their child had been in pain due to their dental problem within the last month. Additionally, RTT significantly predicted the number of sleepless nights due to the child’s dental problem. This indicates the longer a child had to wait, the greater their affected sleep that could lead to further problems, for example, performance at school, or in their school readiness for younger children [21]. This also emphasises the importance of pain and sepsis management during the ensuing wait for DGA.

While dental pain was reported by two thirds of respondents other negative effects were less prevalent, with approximately a fifth of respondents reporting issues around chewing or talking and effects on emotion, in particular with irritability. However, the majority of respondents (78%) reported their child had experienced at least one of the impacts listed. Therefore respondents had been negatively affected by their dental problem, which could have been exacerbated by a lengthy RTT. One area this research explored, which went beyond what is normally asked in the Child Dental Health Survey, was if the child had time off from school and how many days this had been. Approximately a quarter of school aged children (26%) had missed school with an average of three school days missed due to their dental problem. This should be considered alongside the fact that many children would be missing additional school days while attending the hospital and recovery the following day culminating in the majority of children missing at least 2 school days with some children being absent for up to 15 days. These missed school days will not only effect the child, but the family as well. Forty one percent of all carers whose children were absent from school were employed and this could have resulted in days lost from work; for the missed school days, any pre-assessment visit, the day of the procedure and perhaps for several days after before the child was ready to return to school.

An additional troubling element emerging from the analysis is the high number of repeat DGAs seen across the hospitals, particularly for hospital 5 and 6 (that served children from the same community). This impacts on the number of DGAs seen overall and builds pressure on this service, increasing the potential wait in different regions. Previous research has indicated that once a child develops caries it can develop rapidly and consequently children with dental caries should be considered differently from those caries free children [22]. Even when carious teeth have been removed following a GA, given the high number of repeat GAs there is evidence to suggest that children carry the legacy of the disease with them, potentially due to the fact that the causative factors have not been addressed [23, 24]. This could include children not being taken for preventative, routine care or receiving timely treatment. Previous research indicates that those who receive DGA do not always respond to simple preventative messages [25, 26] although parents have stated they would welcome a variety of health care interventions at this stage to avoid repeat DGA’s in the future [23]. The repeat GAs suggest more prevention needs to be done both on the lead-up to GA extraction and afterwards as a proportion of children may well return even after all decayed teeth are removed.

Treatment of carious teeth in children can be an emotive and contentious issue. While guidelines are in place [27] it is up to the referring and then operating dentists to select from the options available for each child. Choices need to be made between restorations or extraction, and between care in either primary or secondary settings. There are uncertainties around which are the best methods to treat carious teeth in young children. One retrospective study of clinical records suggested that the restoration of teeth in general dental practice did not necessarily prevent pain and extraction from occurring [28]. This idea has been continued further with current research exploring whether filling a tooth is the most effective method of managing caries in primary teeth [29].

The conflicts in treatment advice could potentially add to, and put pressure on DGA services if dentists refer when another treatment option is possible. If a proportion of children could be seen and treated in an alternative setting i.e. using other anxiety control measures and not referred for GA, the overall wait time for those children for whom a GA is essential will be reduced. The use of various treatment options may be one of a variety of reasons for the varying wait times observed across regions which are then seen to impact on a child's life in a variety of ways i.e. pain, sleepless nights, etc. It should be noted for some children, who are too young, anxious, unable to co-operate or have relevant medical conditions, which prevent treatment being offered in another way, DGA extraction may be the only viable option for dental treatment.

## Conclusion

Our data suggest the need for effective management of pain and sepsis while children await DGA. Improved clinical management and prevention of caries within primary care could reduce the number of children being referred for GA extraction. This would also have the effect of reducing wait times for those children for whom no other option is available except DGA. Consideration should be given not only to the number of children referred needing a GA but also the high number of repeat GAs. Considering the concept of “making every contact count” there may be opportunities to deliver preventive advice, or even treatment (such as fissure sealants) during DGA appointments to positively impact on oral health and in reduce future extraction under GA.

## Abbreviations

GA: General anaesthetic
DGA: Dental general anaesthetic
RTT: Referral to treatment.
